# Dangerousness: T-wave pseudonormalization in a patient with Wellens’ syndrome: A case report

**DOI:** 10.1097/MD.0000000000041176

**Published:** 2024-12-27

**Authors:** Meixian Lei, Mingqing Yuan, Ling Gao

**Affiliations:** a Jiujiang City Key Laboratory of Cell Therapy, Jiujiang No. 1 People’s Hospital, Jiujiang, Jiangxi, China; b Department of Cardiology, Jiujiang No. 1 People’s Hospital, Jiujiang, Jiangxi, China.

**Keywords:** myocardial, Wellens’ electrocardiogram pattern, Wellens’ syndrome

## Abstract

**Rationale:**

Wellens’ syndrome electrocardiogram (ECG) pattern consists of symmetrically inverted (or biphasic) T waves in the precordial leads, frequently in V2–V3, which is associated with critical stenosis of the left anterior descending (LAD) coronary artery and impending myocardial infarction. Timely diagnosis and early treatment of Wernicke’s syndrome are of utmost importance. Here, we present the clinical characteristics and treatment outcomes of patients with Wellnes’ syndrome.

**Patient concerns:**

A 62-year-old male presented with intermittent chest pain for 6 days while resting, accompanied by chest tightness and sweating. On admission, the patient had no chest pain, vital signs were stable, and physical examination revealed no positive findings. ECG after admission revealed a normal sinus rhythm with poor progression of R waves in the precordial leads. Blood count, biochemical tests, and cardiac biomarkers were all within normal ranges. The patient’s ECG before admission revealed biphasic T waves in leads V2–V6.

**Diagnoses and Interventions:**

Dual antiplatelet, nitrate, and statin drugs were administered and the patient underwent urgent coronary angiography. The results revealed that the proximal LAD coronary artery was nearly completely occluded. Intravascular ultrasonography confirmed plaque rupture with thrombosis in the proximal LAD artery, with a minimum lumen area of 2.4 mm^2^. The patient was diagnosed with Wellens’ Syndrome. A drug-eluting stent was successfully implanted following balloon dilatation.

**Outcomes:**

The left ventricular ejection fraction and reexamination levels of troponin T and B-type natriuretic peptides were normal after the operation. The patient was discharged 6 days later.

**Lessons:**

Enhancing physicians’ awareness of the electrocardiographic patterns associated with Wellens’ syndrome facilitates the early identification of this condition, enabling the timely initiation of pharmacological and revascularization treatments for acute coronary syndrome. This proactive approach effectively mitigates the risk of acute myocardial infarction in patients and significantly improves their prognoses.

## 1. Introduction

In 1982, De Zwaan et al^[1]^ described Wellens’ electrocardiogram (ECG) pattern as a characteristic of biphasic or inverted T waves in the precordial leads. It was confirmed to be associated with severe stenosis of the proximal left anterior descending (LAD) coronary artery in 1989.^[2,3]^ The duration of biphasic or inverted T-waves associated with Wellens’ syndrome can range from hours to weeks. It can also rapidly progress to acute extensive anterior ST-segment elevation myocardial infarction.^[4,5]^ Emergency coronary angiography and early revascularization are critical in patients with Wellens’ syndrome, and cardiac stress tests should not be performed.

## 2. Case report

**Figure 1.**
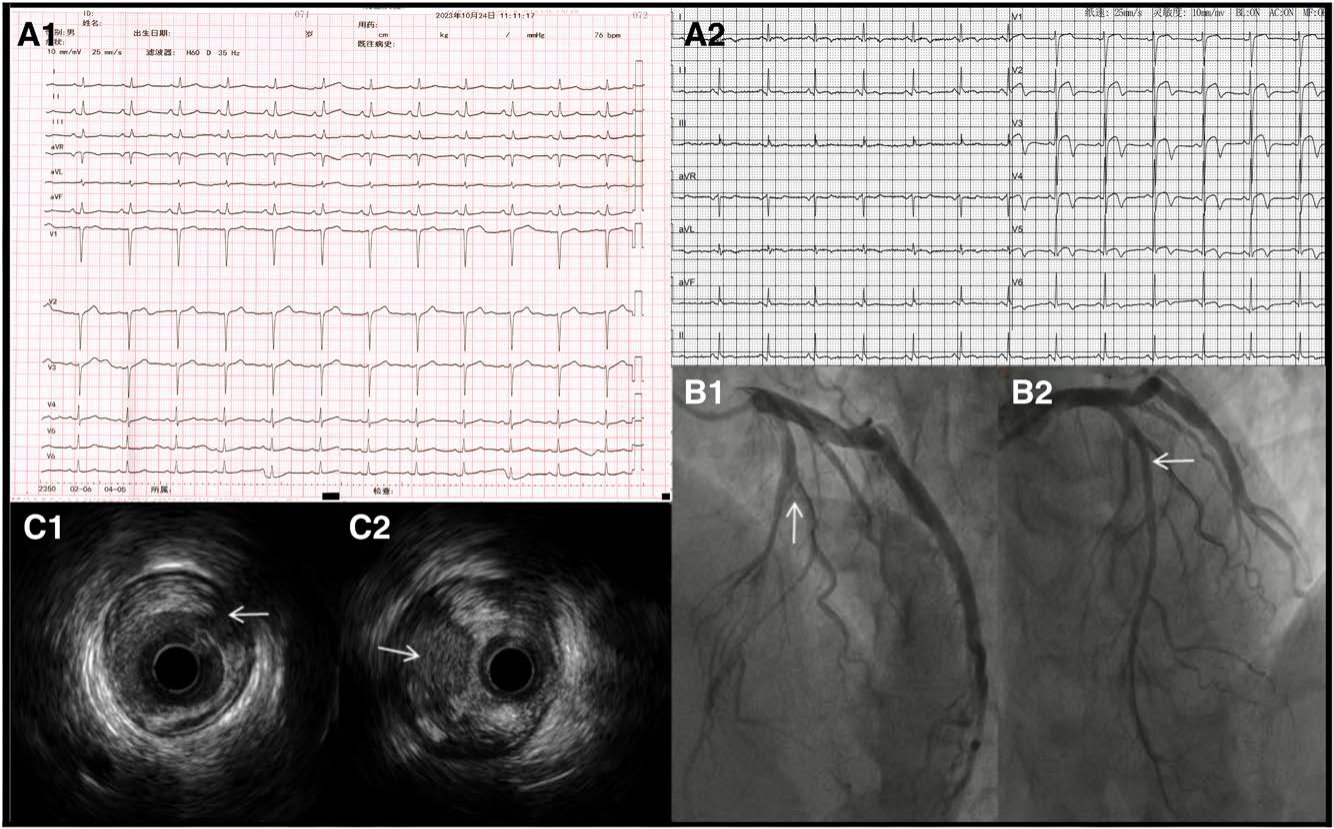
(A) The ECG before and after admission (speed 25 mm/s, 10 mm/mV). ECG = electrocardiogram.

A 62-year-old male presented with intermittent precordial chest pain at rest for 6 days, accompanied by chest tightness and sweating. Benazepril was administered to the patients with a history of hypertension. At presentation, the patient was hemodynamically stable. His vital signs included a blood pressure of 112/76 mm Hg, pulse rate of 78 beats per minute, respiratory rate of 14 breaths per minute, temperature of 36.2°C and oxygen saturation of 100% in room air. He had clear lungs, and his heart sounds were consistent without murmurs or gallops. The results of other systemic examinations were normal. ECG examination immediately after admission revealed normal sinus rhythm, normal heart rate, and poor progression of R-waves in the precordial leads (Fig. 1, panel A1). The patient underwent ECG assessment in the outpatient department prior to admission. Sinus rhythm with biphasic T waves in leads V2 to V6 (Fig. 1, panel A2) was observed on the first ECG. The results of complete blood count and blood chemistry tests were within acceptable ranges. His myoglobin level was 40 ng/mL (reference range, <70 ng/mL), his creatine kinase isoenzyme level was 12 U/L (reference range, 0–25 U/L), his cardiac troponin T level was 0.01 ng/mL (reference range, <0.1ng/mL), and his B-type natriuretic peptide level was 20.21 pg/mL (reference range, <125pg/mL). In addition to intermittent precordial chest pain, ECG changes, and normal cardiac serum markers, Wellens’ syndrome was considered. Dual antiplatelet, nitrate, and statin drugs were administered and the patient underwent urgent coronary angiography. The results revealed that the proximal LAD artery was nearly completely occluded (Fig. 1, panel B1). Intravascular ultrasonography confirmed plaque rupture with thrombosis in the proximal LAD artery (Fig. 1, panels C1 and C2), and the minimum lumen area was 2.4 mm^2^. A drug-eluting stent was successfully implanted after balloon dilatation (Fig. 1, panel B2). Echocardiography revealed no abnormal left ventricular wall motion after the procedure, normal left ventricular ejection fraction (65.1%), and normal reexamination levels of troponin T and B-type natriuretic peptides. The patient was discharged 6 days later.

## 3. Discussion

In 1982, De Zwaan et al^[1]^ initially described a pattern of characteristic ECG changes in the anterior leads, suggesting significant stenosis of the proximal LAD. They performed a prospective study involving 180 patients with unstable angina and further confirmed that this ECG pattern is 100% associated with significant proximal LAD disease on cardiac coronary angiography.^[2]^ Among these patients, 33 had complete occlusion of the LAD artery, whereas 147 presented with mean narrowing of 85%. There were 53 (29%) and 97 (54%) patients with occlusion or stenosis located proximal to the first septal perforator and between the first and second septal branches, respectively.^[2]^

These clinical characteristics and ECG patterns, currently known as Wellens’ syndrome, are as follows: unstable angina; symmetric and deeply inverted (type B) or biphasic (type A, initially positive followed by negative deflection from baseline) T waves in leads V2 and V3, occasionally in leads V1, V4, V5, and V6; isoelectric or minimally elevated (1 mm) ST segment; absence of pathological precordial Q waves; absence of precordial R waves; ECG findings present during the pain-free period; and minimal or absent elevation of cardiac serum markers.^[6,7]^ Type A and Type B cases accounted for 24% and 76% of Willens cases, respectively.^[2]^

The pathophysiologic mechanism associated with the ECG pattern of the Wellens’ remains ambiguous. Myocardial interstitial edema creates inhomogeneity in intramyocardial repolarization. Dynamic negative T waves are likely to reflect transient inhomogeneity and dispersion of repolarization caused by edema. Myocardial edema may be responsible for the Wellens’ ECG pattern,^[8]^ as indicated by an increase in the signal intensity of the LV myocardium on T2-weighted sequences of cardiac magnetic signals observed in individual studies.^[9]^

T-wave inversions associated with Wellens’ syndrome are not a sign of acute LAD artery occlusion, but rather a consequence of LAD reperfusion occurring several hours to days after myocardial ischemia subsides.^[10]^ A study by De Zwaan et al^[1]^ revealed that among 12 patients, 16 who did not undergo bypass surgery experienced significant anterior wall myocardial infarction over an average of 8.5 days, even though symptoms disappeared and the ECG returned to “normal” in some patients. Cardiac stress tests should not be performed in patients with Wellens’ syndrome, as they may be fatal because they may lead to infarction by increasing the myocardial oxygen demand.^[11]^ The symptoms of Wellens’ syndrome in the patients who underwent LAD coronary artery bypass grafting disappeared.

Diagnosing Wellens’ syndrome can be challenging and requires a high level of suspicion. Biphasic T waves are a type of Wellens’ syndrome that is relatively uncommon, subtle, and underappreciated, and may be a more lethal variant. In contrast, the differential diagnosis for the more common “LAD coronary T-wave syndrome” is broad, encompassing left ventricular hypertrophy, myocarditis, pulmonary embolism, cerebral hemorrhage, coronary vasospasm, and Takotsubo cardiomyopathy.^[12–15]^ ECG changes in WS are usually observed during the pain-free interval and often involve cardiac markers that are normally to slightly elevated.

## 4. Conclusion

The Wellens’ syndrome is linked to severe stenosis of the proximal LAD artery. As a sign of spontaneous reperfusion of an occluded LAD artery, patients with Wellens’ syndrome have a high risk of anterior wall myocardial infarction.^[3]^ Physicians should be vigilant regarding this type of ECG and promptly diagnose it. Urgent coronary angiography and early interventional revascularization can prevent anterior wall myocardial infarction.

## Author contributions

**Data curation:** Mingqing Yuan, Ling Gao.

**Methodology:** Mingqing Yuan.

**Visualization:** Ling Gao.

**Writing – original draft:** Meixian Lei.

**Writing – review & editing:** Meixian Lei.
